# Behavioral profile predicts ethanol preference in adolescent mice, but not in adults: A machine learning approach

**DOI:** 10.1111/acer.70203

**Published:** 2026-01-23

**Authors:** Liana C. L. Portugal, Bruno da Silva Gonçalves, Emily de Assis Fagundes, Maria Fernandes Freire de Sá, Cláudio Carneiro Filgueiras, Ana Carolina Dutra‐Tavares, Alex C. Manhães, Yael Abreu‐Villaça, Anderson Ribeiro‐Carvalho

**Affiliations:** ^1^ Laboratório de Neurofisiologia, Departamento de Ciências Fisiológicas, Instituto de Biologia Roberto Alcântara Gomes, Centro Biomédico Universidade do Estado do Rio de Janeiro (UERJ) Rio de Janeiro Brazil; ^2^ Departamento de Ciências Biomédicas e Saúde, Instituto de Biologia Roberto Alcantara Gomes Universidade do Estado do Rio de Janeiro (UERJ) Cabo Frio Brazil; ^3^ Departamento de Ciências Faculdade de Formação de Professores da Universidade do Estado do Rio de Janeiro São Gonçalo Brazil

**Keywords:** adolescence, ethanol, individual differences, machine learning

## Abstract

**Background:**

Clinical studies suggest that adolescents display a complex behavioral profile characterized by traits that increase their susceptibility to alcohol experimentation and impaired control over use. In the present study, we applied a machine learning model to predict the impact of diverse behavioral phenotypes on ethanol preference during adolescence and adulthood in mice.

**Methods:**

C57BL/6 and Swiss mice were assigned to one of two age groups: adolescents (starting at PN40) or adults (starting at PN120). Over the next 3 days, novelty‐seeking, anxiety‐like behavior, sociability, coping behavior, and natural reward response were evaluated using the following behavioral tests: hole‐board, elevated plus maze, three‐chamber sociability, forced swimming, and sucrose preference, respectively. During the subsequent 5 days, alcohol preference behavior was assessed using the two‐bottle choice paradigm (10% ethanol). We trained machine learning regression models to predict alcohol preference in each age group.

**Results:**

The analysis model significantly predicted ethanol preference based on behavioral phenotypic profiles in mice during adolescence, but not in adulthood. Notably, the behavioral traits that contributed most to the prediction were sucrose preference and sociability time. Sucrose preference had a positive predictive value. Distinctively, sociability time had a negative predictive value, indicating an inverse relationship with ethanol preference.

**Conclusions:**

These findings suggest that behavioral phenotypes during adolescence, particularly natural reward sensitivity and sociability, are key predictors of ethanol preference. The negative association between sociability and alcohol intake highlights the potential protective role of social interaction. The absence of predictive value in adulthood underscores adolescence as a critical developmental window during which behavioral traits may influence vulnerability to alcohol use.

## Graphical abstract

The present study reveals that adolescent behavioral phenotypes strongly predict ethanol preference in mice, while no predictive patterns emerged in adulthood. Machine learning analysis identified sucrose preference as a positive predictor and sociability as a negative predictor of alcohol intake, highlighting natural reward sensitivity and social interaction as pivotal factors. These results underscore adolescence as a critical developmental stage where behavioral traits shape vulnerability to alcohol use.

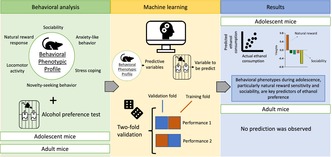

## INTRODUCTION

During adolescence, the brain continues to undergo fundamental developmental processes. Synaptic pruning, reduction of cortical gray matter volume, and myelination are key developmental features of this period (Paus et al., 2008). These changes in the adolescent brain shape a behavioral profile that facilitates the transition to becoming a self‐sufficient member of society. However, adolescence is also marked by an increased incidence of several psychiatric disorders, including substance abuse and dependence (Costello et al., 2003). Clinical studies suggest that adolescents exhibit a complex behavioral profile composed of some behavioral traits that heighten their susceptibility to drug experimentation and loss of control over use (Comeau et al., 2001; Woicik et al., 2009). Traits such as social behavior, heightened responses to positive rewards, and a propensity for risk‐taking and novelty‐seeking have been associated with drug use (Adalbjarnardottir & Rafnsson, 2002; Geier, 2013). Additionally, anxiety levels and the ability to cope with stress have been identified as relevant factors influencing substance use and abuse in adolescents (Comeau et al., 2001).

Alcohol use typically begins during adolescence, and it has become one of the most widely used and abused substances among individuals in this age group worldwide (Spear, 2015). Adolescents are particularly vulnerable to the neurotoxic effects of alcohol (for a review, see Crews et al., 2007) and consumption during this critical stage of development impairs prefrontal function, leading to a decline in executive control and decision‐making abilities (Carbia et al., 2018; Squeglia et al., 2015). This, in turn, increases vulnerability to various adverse consequences, including engaging in unsafe sexual practices, driving under the influence of drugs, adopting violent behaviors, and involvement in criminal activities (Caamano‐Isorna et al., 2021; Hingson, 2010; Swahn et al., 2004). Additionally, early ethanol consumption increases the risk of developing alcohol use disorder in adulthood (Addolorato et al., 2018). The susceptibility to alcohol consumption and eventual abuse may be influenced by biological and cultural factors, reflecting the long‐standing nature versus nurture debate. Given the complexity of human populations, preclinical research is necessary to establish the causal mechanisms underlying the relationship between behavioral traits and substance use (Spear, 2016). Animal models help rule out cultural influences in this association while providing insights into the underlying biochemical processes. In this regard, such models offer new possibilities for developing more effective interventions for alcohol use disorder.

The majority of preclinical studies on individual differences in the effects of drugs of abuse have primarily focused on substances, such as amphetamines and cocaine (Falco & Bevins, 2015), often in adulthood. However, some studies have investigated the potential risk behaviors associated with ethanol preference in rodents. For example, locomotor activity has been positively correlated with ethanol self‐administration under certain conditions in adult rats (Nadal et al., 2002; Wingo et al., 2016). In another study, a positive correlation was observed between saccharin and ethanol intake in genetically heterogeneous adult rats (Overstreet et al., 1993). Sociability can also influence alcohol preference in animal models. Varlinskaya et al. (2015) found that highly sociable males and socially anxious females exhibit increased alcohol consumption in social settings. Adolescent social isolation may also lead to higher ethanol intake in male rats (Skelly et al., 2015). However, findings on the impact of social isolation on alcohol use have been inconsistent, with some studies reporting no change or even a decrease (for a review, see Anacker & Ryabinin, 2010). Similarly, novelty seeking, typically assessed by measuring activity in new environments, has shown mixed associations with alcohol intake: positive (Karatayev et al., 2010), negative (Gingras & Cools, 1995), or none (Hayton et al., 2012). Anxiety is one of the most studied behavioral traits in relation to susceptibility to ethanol intake. However, findings in the literature also remain inconsistent. Depending on the rodent strain, higher levels of anxiety have been associated with increased, decreased, or unchanged ethanol consumption (da Silva et al., Da Silva et al., 2004; Pelloux et al., 2015). These inconsistencies may stem from the influence of diverse behavioral profiles that can alter ethanol preference patterns. In other words, besides interindividual variability, each strain exhibits varying levels of novelty‐seeking behavior, anxiety, stress responses, and other behavioral phenotypes, making the analysis based on isolated behaviors unreliable. A more precise assessment of individual differences in susceptibility to ethanol preference can be achieved by examining multiple behavioral phenotypes under the same conditions. Moreover, there is a notable lack of studies evaluating the relative contribution of multiple behavioral traits on ethanol preference, particularly in elucidating the greater susceptibility of adolescents compared with adults.

Currently, artificial intelligence, particularly machine learning approaches, can be applied to the analysis of behavioral data in animal studies (for a review, see Valletta et al., 2017). Machine learning is a rapidly evolving field that has the potential to identify multivariate patterns in the data that enable the classification (classification model) or the prediction of continuous variables (pattern regression model) at the individual subject level. In the context of machine learning, the term “predict” means that, once the model has learned a relationship between a set of phenotypic profiles and labels (e.g., ethanol preference), given a new pattern (unseen data), it can predict its label. The goal of the predictive model was to generalize effectively, meaning it should make accurate predictions based on previously unseen data. Here, we used machine learning (pattern regression) to integrate multiple behavioral variables to predict ethanol preference. It is important to clarify that prediction can also be addressed using a more conventional approach (e.g., univariate linear and logistic regression) to build mathematical descriptions of the data. However, the latter approaches differ from machine learning in their rationale; conventional approaches start with an assumption about the underlying data distribution, and the models are typically constrained by strong assumptions. The focus of conventional approaches is on inference, estimating the parameters of the model that fits the observed data, whereas for machine learning, the focus is typically on prediction, learning this hypothesis directly from the training dataset. This hypothesis‐free approach makes machine learning an attractive choice to integrate multiple behavioral variables to predict ethanol preference. Besides the aforementioned innovative dimension, machine learning advantages include the following: (1) the capability to evaluate relationships among multiple variables simultaneously; (2) the effectiveness in identifying patterns in complex datasets; and (3) a lack of constraint by traditional assumptions, such as a normal distribution of the data or an a priori model (Orrù et al., 2020; Valletta et al., 2017). In this study, we aim to apply pattern regression models to predict the impact of multiple behavioral phenotypes on ethanol preference in the two‐bottle choice paradigm during adolescence and adulthood, using inbred (C57BL/6) and outbred (Swiss) mice.

## MATERIALS AND METHODS

All experimental procedures were approved by the Institute of Biology/UERJ Ethical Committee for Animal Research (protocol: CEUA/0022021) and conducted in accordance with Brazilian Law No.: 11.794/2008. Efforts were made to minimize the number of animals used and to prevent animal suffering. To scope the dimension of the external validity and translational relevance of the data, we included both an inbred (C57BL/6) and an outbred (Swiss) strain. The genetic homogeneity of inbred strains reduces interindividual variability, while the genetic diversity of outbred strains better reflects natural population variability. All Swiss and C57BL/6 mice were bred and housed in our animal facility under controlled conditions (21–22°C) on a 12‐h light/dark cycle (lights on at 2:00 a.m.). Both strains were obtained from a colony maintained at the Universidade Federal Fluminense (Niterói, Brazil) for more than 60 generations. Food and filtered water were provided ad libitum. All animal manipulations and behavioral tests were conducted in a sound‐attenuated room adjacent to the animal facility.

Animals were weaned on postnatal day 23 (PN23). From then on, they were housed in same‐sex groups of two to three per cage, with free access to food (standard laboratory diet for rodents, Nuvilab–NUVITAL Nutrientes LTDA, Paraná, Brazil) and water. They were assigned to one of two experimental age groups: adolescents (beginning at PN40) or adults (beginning at PN120). At the start of the experimental period, animals were individually housed in standard cages (30 × 20 × 13 cm) without environmental enrichment. To minimize inconsistencies in the literature concerning the onset and end of adolescence, we selected a timeframe representing mid‐adolescence (PN40) alongside an age unequivocally classified as mature adulthood (PN120) (Spear, 2000). On the first day, novelty‐seeking behavior was assessed using the hole‐board test (9:00–11:00 a.m.) and anxiety‐like behavior was evaluated in the elevated plus maze test (11:00–1:00 p.m.). On the following day, sociability was evaluated by the three‐chamber sociability test (11:00 a.m.–1:00 p.m.) and followed by the forced swimming test (2:00–4:00 p.m.). The forced swimming test was used to assess coping strategies for acute inescapable stress. All test variables were scored using video recordings, and observers were blind to the age of the mice. The raters underwent a standardized training process to demonstrate high reliability in their evaluations. To ensure accuracy, their assessments were compared with a gold‐standard evaluation. Only those who achieved a correlation coefficient greater than 0.95 in the reliability analysis were considered qualified to proceed with the experimental assessments. Following these assessments, the natural reward response was evaluated in the sucrose preference test for 24 h. In the subsequent 5 days, alcohol drinking behavior was examined using the two‐bottle choice paradigm. To minimize the effects of multiple testing, the order of the behavioral tests was defined by the assumed degree of stress of each test (from lower to higher). The sucrose preference test was last due to its similarity in paradigm to the two‐bottle choice test.

### Behavioral tests

All test procedures are described in detail in previous publications (Demarque et al., 2020; Ribeiro‐Carvalho et al., 2011, 2020). In the hole‐board test, each animal was placed for 5 min in an enclosed arena divided into 16 equal‐sized rectangles, each containing a hole in the center. The number of head dips into the holes was recorded as a measure of novelty‐seeking behavior (Abreu‐Villaça et al., 2006). The elevated plus maze consists of a plus‐shaped apparatus with two open arms (5 × 28.5 cm, no walls) and two enclosed arms (5 × 28.5 × 14 cm, enclosed by walls), arranged perpendicularly and elevated 50 cm above the floor. The animal was placed in the center of the maze, facing an enclosed arm, and allowed to explore for 5 min. The percentage of open‐arm entries (%Entries OA: number of entries into open arms divided by the total number of entries into both open and enclosed arms) was used as a measure of anxiety‐like behavior, with higher %Entries OA indicating lower anxiety levels and vice versa. The number of closed‐arm entries (Entries CA) was recorded as a measure of overall activity. The three‐chamber sociability apparatus consists of a rectangular, transparent plexiglass box divided into three chambers by two plexiglass walls with openings. Each side chamber contains a cylindrical grid‐bar enclosure. In one of these enclosures, named as the social chamber, an “unfamiliar” mouse (same sex and age as the experimental subject) was placed, while the other enclosure remained empty. The social chamber was randomly assigned. The experimental mouse was first habituated in the middle chamber for 5 min, after which it was allowed to explore the entire apparatus for 10 min. Sociability was measured as the percentage of time spent in the social chamber relative to the total time spent in both the social and empty chambers. In the forced swimming test, each mouse was placed in a plastic container (diameter: 21 cm, height: 23 cm) filled with 16 cm of water maintained at approximately 25°C. Climbing behavior (time in seconds), defined as active, vigorous movements of the forepaws against the walls was measured. For the sucrose preference test, mice were individually housed and given a free choice between two graduated bottles for 24 h—one containing a 2% sucrose solution (Sigma Aldrich) and the other containing filtered tap water. Sucrose solution consumption varies as a function of its concentration, progressively increasing with higher concentrations. Here, we used this concentration (2%) aiming to maximize inter‐individual variability and to avoid ceiling effects associated with high concentrations, which would reduce the discriminative power of the measure (Ribeiro‐Carvalho et al., 2011). Individual consumption was calculated as the difference between the initial volume provided and the remaining volume after 24 h of access. To ensure accuracy, values were corrected for potential losses due to leakage by including control bottles placed in empty cages under identical conditions and subtracted from fluid consumption data. The percentage of sucrose consumption was calculated as follows: ((sucrose intake/total intake) × 100).

### Two‐bottle choice test paradigm

In this paradigm, animals were given a free choice between two bottles: one containing a 10% ethanol solution (Sigma Aldrich) and the other containing filtered tap water (Peirce et al., 1998). The free‐choice period lasted from PN43 to PN47 for adolescents and from PN123 to PN127 for adults. Bottles were cleaned and refilled daily, and their positions were switched each day to prevent side preference bias. Individual consumption was calculated as the difference between the initial volume provided and the remaining volume after 24 h of access. Fluid loss due to leakage was also accounted for by placing two identical bottles in an empty cage (“blank”) and subtracting this value from the recorded fluid consumption data. The percentage of ethanol preference (%Etoh) during the two‐bottle choice test was calculated as follows: %Etoh = ethanol consumption/(ethanol consumption + tap water consumption) * 100.

### Statistical analysis

Data were compiled and shown as means and standard errors. Initially, univariate analysis of variance (ANOVA) was conducted on each behavioral measure, including novelty seeking, anxiety, motor activity, sucrose preference, sociability, and stress coping, as well as on ethanol preference (variable to be predicted). These analyses were performed separately totaling seven independent ANOVAs. Sex (male or female), strain (Swiss or C57BL6), and age (adolescence or adulthood) were considered as factors in these analyses. All statistical analyses were carried out using the JASP (version 0.18.3) software, and a significance level of *p* = 0.05 was adopted for all analyses.

### Pattern regression analysis

The pattern regression analysis was implemented in PRoNTo (version 03, Schrouff et al., 2013) to predict ethanol preference based on behavioral phenotypic profiles in Swiss and C57BL/6 mice during both adolescence (Model 1) and adulthood (Model 2). In this study, we applied two algorithms to predict ethanol preference: Gaussian Process Regression (GPR) and Kernel Ridge Regression (KRR). Specifically, GPR is a probabilistic regression approach developed by Williams and Rasmussen (2006) and KRR is an algorithm based on kernel functions that uses a squared error loss function for the regularization parameter (Hoerl & Kennard, 1970). The performance between the two algorithms showed no significant differences. Thus, for brevity, only the GPR results have been included in the main manuscript (results for the KRR model can be found in the Table S1).

**FIGURE 1:**
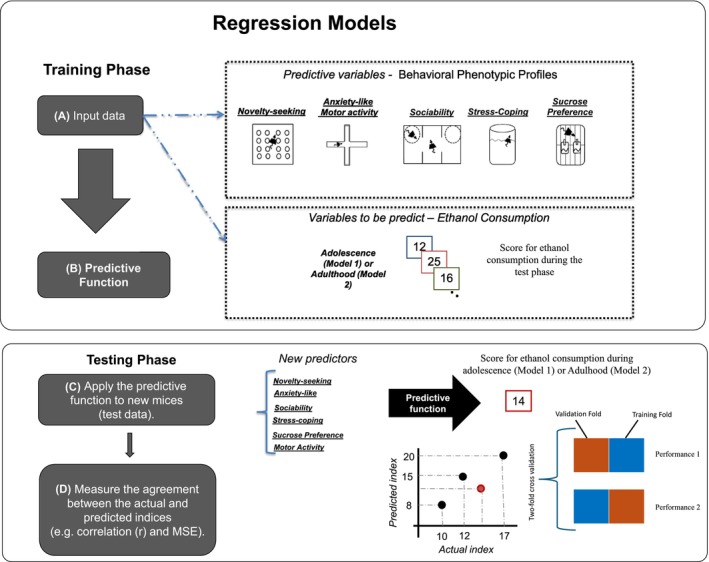
Regression models: The process of constructing pattern regression models comprises two stages: Training and testing. Superior panel: Training phase: (A) The training data for the Gaussian Process Regression (GPR) regression model consists of examples that pair the behavioral phenotypic profiles of each animal and the corresponding ethanol consumption score. (B) During the training, the GPR model learns the contribution of each behavioral phenotypic profile for the predictive function. Inferior panel: Testing phase: (C) During the testing phase, given the behavioral phenotypic profile of a test mice, the GPR model predicts its corresponding clinical score. (D) The model performance is evaluated using metrics that measure the agreement between the predicted and actual clinical scores: Pearson's correlation coefficient (*r*) and normalized mean squared error (NMSE). (E) The twofold cross‐validation involves dividing the data into two disjoint folds (Model 1 *n* = 23 animals in each fold and Model 2 *n* = 40 animals in onefold and 39 in the other fold). Data from one set are left out as test samples (red square), and data from the other set are used to train the model (blue square). This procedure is then repeated so that each dataset is left out once for testing.

Briefly, the process of constructing pattern regression models comprises two stages: training and testing. The dataset is partitioned into training and testing sets (as explained in the next section, the number of animals in each set depends on the cross‐validation scheme chosen). During the training phase, the model is then trained to learn the association between the phenotypic behavioral profiles and a label (as behavioral phenotypic profile: novelty seeking, anxiety, locomotor activity, sociability, stress coping and sucrose preference, as label: ethanol preference). Once the model had “learned” this association (i.e., the model parameters were estimated based on the training data), it could be used to predict the label of a new test example (unseen data). This procedure is repeated a number of times according to the cross‐validation scheme chosen and the results are averaged across these repetitions. We trained one model for adolescence and another for adulthood. In addition, we applied the operation to mean center the features using the training data. The output of the model is the predicted ethanol preference score obtained during the test phase which is then compared with the actual value of the ethanol preference. Please see Figure 1 for more details.

### Cross‐validation strategies

To evaluate the GPR performance, we used two different *cross‐validation* strategies: twofold cross‐validation and fivefold cross‐validation. The twofold cross‐validation involves dividing the data into two disjoint folds (Model 1 *n* = 23 animals in each fold and Model 2 *n* = 40 animals in onefold and 39 in the other fold). Data from one set are left out as test samples and data from the other set are used to train the model. This procedure is then repeated so that each dataset is left out once for testing. The fivefold cross‐validation involves dividing the data into five disjoint sets. Data from each set are left out once for testing and data from the remaining four sets are used to train the model. This procedure is then repeated five times so that each set is left out once. It is important to emphasize that we used a fivefold cross‐validation strategy to demonstrate that the results were not dependent on a specific cross‐validation scheme (results for the fivefold cross‐validation strategy can be found in the Table S1). Because our sample size in Model 1 (*n* = 46) was not divisible by five, fourfolds contained 10 animals each, while the remaining fold contained 6 animals. The same occurred in Model 2 (*n* = 79); fourfolds contained 16 animals each, while the remaining fold contained 15 animals. It is important to emphasize that we manually balanced the proportion of animals' strains and sex across the different folds.

### Performance of the model

The performance of the pattern regression models was measured using two metrics of agreement between the predicted and actual scores: correlation coefficient (*r*) and normalized mean squared error (NMSE) (Portugal et al., 2019). The correlation coefficient (*r*) describes the strength of the linear relationship between two variables. A small correlation indicates poor predictive performance. MSE is the mean of the squared differences between the predicted and true scores; it represents the mean error between the predicted and actual scores, and is commonly used to evaluate the performance of predictive models. MSE was normalized by dividing the MSE by the variance in the target values (NMSE). The significance of the regression performance measures was determined using nonparametric permutation tests; that is, the same cross‐validation procedure described above was performed 1000 times with the labels permuted across the participants. The *p*‐value was calculated by counting how many times the absolute value of the metric with the permuted labels was equal to or greater (less for MSE) than the absolute value of the metric obtained with the correct labels and dividing by 1000. The results were considered significant when the model performed equal to or better than the model without shuffling the labels for at most 5% of the time across 1000 permutations (Schrouff et al., 2016).

### Covariate

Sex and strain (Swiss × C57BL/6) were considered as potential confounders that could affect the pattern regression models. However, removing confounds associated with the variable we want to predict is not recommended because this adjustment is likely to remove not only the variability in the data due to the confounds but also the variability in the data associated with the labels (Rao et al., 2017). Therefore, we used two‐sample *t*‐tests to determine whether sex and strain were systematically related to ethanol preference. For Model 1 (adolescence), neither sex (*p* = 0.57, Female: mean = 46.4, SD = 21.9, Male: mean = 42.3 SD = 22.4) nor strain (*p* = 0.61; Swiss: mean = 42.7, SD = 19.0, C57BL/6: mean = 46.2 SD = 25.2) differed significantly in ethanol preference. As both covariates were not systematically related to ethanol preference, we included sex and strain as covariates/confounders in Model 1 for pattern regression analyses, using an approach that accounts for the training and testing separation, as described by Rao and Mourao‐Miranda (2017). For Model 2, males and females differed significantly in ethanol preference (*p* = 0.05, Female: mean = 56.8, SD = 27.0, Male: mean = 44.0 SD = 28.7) and Swiss mice had a tendency to differ from C57BL/6 mice in ethanol preference (*p* = 0.06, C57BL: mean = 62.4, SD = 20.4, Swiss: mean = 52.3 SD = 25.9). Due to these observed associations, we did not include sex and strain as covariates/confounders in Model 2.

### Model interpretation

The weights represent the contribution of each behavioral phenotypic profile to the linear predictive function and can be explicitly computed and plotted for interpretation and discussion. As previously discussed in the literature (Schrouff et al., 2018), the weights of linear machine learning models cannot be thresholded to make specific inferences as in classical (univariate) techniques. Since each cross‐validation fold yields a different weight vector, the final psychometric weight is the average across the folds divided by its Euclidean norm. For the sake of brevity, we illustrate only the twofold cross‐validation in the manuscript.

## RESULTS

### Behavioral tests

All main effects and interactions are shown in Table 1. A significant main effect of age was observed in the hole board (*F* = 17.8, df = 1, *p* < 0.001) as well as in the activity measures of the elevated plus maze (*F* = 18.9, df = 1, *p* < 0.001). As indicated by the ANOVAs, adolescent mice exhibited greater novelty‐seeking behavior (number of head dips into the holes) and were more active (number of closed‐arm entries) than adult ones. Our data also showed significant main effects of sex (*F* = 4.2, df = 1, *p* = 0.04) and age (*F* = 6.8, df = 1, *p* = 0.01), indicating that female and adolescent animals displayed fewer anxiety‐like behaviors than males and adult animals, respectively. Furthermore, the results of the sociability test revealed significant effects of strain (*F* = 48.5, df = 1, *p* < 0.001), age (*F* = 4.7, df = 1, *p* = 0.033), and a significant interaction between sex and strain (*F* = 8.03, df = 1, *p* = 0.005). According to the data, adult animals and Swiss mice spent more time socializing when compared to adolescent and C57BL/6 mice, respectively. The interaction results indicated that the difference in the duration of socialization between Swiss and C57BL/6 mice was more pronounced in male than in female animals. The results of the forced swimming test revealed a significant main effect of strain (*F* = 16.1, df = 1, *p* < 0.001), indicating that C57BL/6 mice exhibited higher levels of climbing behavior than Swiss mice. Finally, no effects or interactions were observed in sucrose preference. In the case of ethanol preference, the results showed a significant main effect of age (*F* = 9.4, df = 1, *p* = 0.003), indicating that ethanol preference was greater in adulthood than in adolescence. In our model, the average daily ethanol consumption in Swiss mice was 7.5 ± 3.5 g/kg during adolescence and 5.9 ± 3.3 g/kg during adulthood, whereas C57BL/6 mice consumed more ethanol overall (*F* = 18.8, df = 1, *p* < 0.001), with averages of 9.9 ± 5.3 g/kg and 10.3 ± 5.0 g/kg, respectively.

**TABLE 1:** Descriptive and overall analysis of behavior tests.

Variables	Adolescence				Adulthood				ANOVA			
	Swiss		C57BL		Swiss		C57BL					
	Female	Male	Female	Male	Female	Male	Female	Male	Main effect of age	Main effect of strain	Main effect of sex	Interaction
Novelty seeking	135.5 (15.2)	144.4 (17.1)	115.2 (10.0)	129.1 (12.5)	80.9 (10.8)	117.7 (21.7)	74.8 (9.6)	82.5 (7.3)	*p* < 0.001	*p* = 0.06	*p* = 0.09	–
Anxiety	12.6 (3.0)	7.1 (1.9)	16.3 (5.3)	9.7 (2.6)	2.4 (1.5)	3.4 (2.6)	11.8 (3.7)	4.2 (1.1)	*p* = 0.01	*p* = 0.07	*p* = 0.04	–
Locomotor activity	9.8 (1.1)	7.8 (1.1)	8.4 (1.0)	8.3 (1.2)	6.4 (1.0)	5.7 (1.1)	4.7 (0.6)	5.7 (0.7)	*p* < 0.001	*p* = 0.35	*p* = 0.47	–
Sociability	63.6 (10.3)	73.9 (7.9)	54.2 (4.7)	26.4 (5.0)	75.5 (7.9)	85.0 (4.8)	47.7 (3.3)	48.8 (2.7)	*p* = 0.03	*p* < 0.001	*p* = 0.78	Sex * Strain *p* < 0.001
Stress coping	80.6 (13.0)	79.2 (10.5)	116.5 (21.9)	79.3 (10.9)	52.9 (7.9)	52.3 (8.0)	93.9 (9.2)	106.4 (10.8)	*p* = 0.13	*p* < 0.001	*p* = 0.42	–
Sucrose preference	56.4 (7.1)	58.0 (9.1)	38.0 (8.5)	47.0 (11.2)	47.8 (6.6)	50.1 (7.0)	62.4 (5.0)	38.9 (6.6)	*p* = 0.99	*p* = 0.23	*p* = 0.61	–
Ethanol preference	50.0 (6.2)	35.4 (4.0)	41.6 (7.7)	49.3 (7.5)	52.7 (5.8)	51.8 (7.2)	64.6 (3.9)	59.3 (4.8)	*p* = 0.003	*p* = 0.15	*p* = 0.44	–

*Note*: Data are expressed by mean (standard deviation). Significant results are displayed in red. Novelty Seeking = number of head dips; Anxiety = %Entries OA; Locomotor Activity = Entries CA; Sociability = % of time spent in the social side; Stress coping = time of climbing behavior (seconds); Sucrose preference = % of sucrose consumption; Ethanol Preference = % of ethanol consumption.

### Pattern regression models

The GPR regression model significantly predicted ethanol preference from behavioral phenotypic profiles in mice during adolescence, but not in adulthood.

### Model 1: Adolescence

**FIGURE 2:**
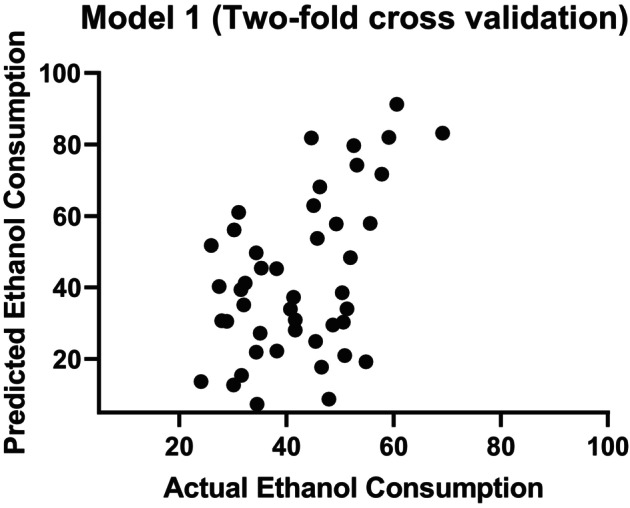
Scatter plots of actual versus predicted values applying a twofold cross‐validation scheme for the ethanol consumption model during adolescence.

**FIGURE 3:**
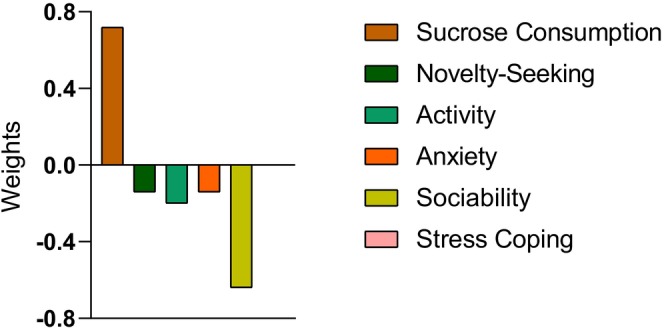
Plot showing the values of the weights for each behavior for the prediction of ethanol consumption during adolescence.

Model 1 consisted of a total of 46 animals, comprising 24 Swiss (12 males and 12 females) and 22 C57BL/6 (13 males and 9 females). When controlling for covariate effects (strain and sex), the correlation coefficient (*r*) and NMSE between the decoded and actual ethanol preference were 0.47 (*p* = 0.004) and 0.74 (*p* = 0.001), respectively (Figure 2 and Table 2). The results for each separate fold can be found in Table S2. Similar results were obtained for ethanol preference without controlling for covariates (*r* = 0.44, *p* = 0.02; NMSE = 0.79, *p* = 0.006). It is important to note that for both cross‐validation frameworks (twofold and fivefold cross‐validation) and for two different algorithms (GPR and KRR), the correlation coefficient and MSE between actual and decoded values were significant (see Tables S1 and S2 for additional data). These findings demonstrate that the results were not dependent on a specific cross‐validation scheme or machine (see Tables S1 and S2 for additional data). We display in Figure 3 the weights indicating the relative contribution of each phenotypic behavioral profile to the GPR for Model 1. Notably, the phenotypic behavioral profiles with the greatest contributions were sucrose preference (0.72) and sociability time (−0.64) and the one that contributed the least was Stress Coping (0.0001). Interestingly, sociability time had a negative predictive value, indicating an inverse association with ethanol preference.

**TABLE 2:** Measures of agreement between actual and decoded scores of ethanol consumption based on behavioral phenotypic profiles.

Models	Measures of agreement	
	*r* (*p*‐value)	NMSE (*p*‐value)
Model 1 adolescence	0.47 (0.004)	0.74 (0.001)
Model 2 adulthood	0.11 (0.27)	1.09 (0.38)

*Note*: Significant results are displayed in red.

### Model 2: Adulthood

Model 2 consisted of a total of 79 animals, comprising 33 Swiss (16 males and 17 females) and 46 C57BL/6 (19 males and 27 females). Due to the observed associations between age and strain with ethanol preference, we do not include age and strain as covariates in Model 2. The correlation coefficient *r* and NMSE between the decoded and actual ethanol preference were 0.11 (*p* = 0.27), and 1.09 (*p* = 0.38), respectively.

Similar results were obtained when we conducted two univariate linear regression models to predict ethanol preference in adulthood and adolescence. As expected, none of the behavioral variables predicted ethanol preference in adulthood. In contrast, during adolescence, sucrose consumption (*β* = 0.46, *p* = 0.004) significantly predicted ethanol preference, while sociability showed a trend toward significance (*β* = −0.28, *p* = 0.054). For details, see Tables S3 and S4.

## DISCUSSION

In the present study, we used a machine learning analysis model to predict the impact of multiple behavioral phenotypes on ethanol preference. Interestingly, the GPR regression model was able to predict ethanol preference based on multiple behavioral phenotypes during adolescence but not in adulthood. Despite the absence of differences in sucrose preference between adolescents and adults, this measure contributed the most to the predictive function in the adolescent model, suggesting a role for the response to natural rewards in ethanol preference at this age. Additionally, sociability levels had a significantly negative predictive value, indicating an inverse relationship with ethanol preference. These findings suggest that animals with low sociability were more likely to exhibit higher alcohol preference. In the following paragraphs, we will discuss the relevance of these findings.

In this study, we used two strains of mice and included both sexes, following NIH recommendations. This approach aims to generate more comprehensive and reliable results, improving the translation of preclinical findings to clinical settings. In the present study, female mice exhibited less anxiety‐like behavior than males, but there was no sex difference in ethanol preference. Additionally, the pattern regression model showed that anxiety‐like behavior had little impact on the increased susceptibility to ethanol preference in adolescent animals. It is possible that sex differences play a significant role in shaping ethanol consumption patterns, particularly in the presence of an underlying mental disorder or an already established alcohol use disorder. In fact, the effects of stress or anxiety on behavioral aspects of relapse, such as craving, are more pronounced in females (for review, see Logrip et al., 2018). However, these findings should be interpreted with caution since all predictive variables contributed to the final prediction. Here, we use Swiss and C57BL/6 strains. Outbred strains, such as Swiss mice, exhibit high genetic heterozygosity, which helps maintain significant genetic diversity within the same colony. In contrast, animals from inbred strains, such as C57BL/6 mice, are assumed to display less trait variability. Our data indicated differences in sociability and forced swimming behavior between these strains. Swiss mice exhibited higher sociability, as shown in a previous study (Mansk et al., 2023), and lower levels of climbing behavior than C57BL/6 mice. In fact, behavioral responses seem to depend on the strain used in the forced swimming test (Bourin et al., 2005). Swiss mice appear to be the most sensitive strain for detecting serotonin‐ and/or noradrenaline‐based antidepressants, while C57BL/6J is the only strain that shows sensitivity to dopaminergic agents in the forced swimming test (Bourin et al., 2005). Despite this, and in accordance with Tuttle et al. (2018), we failed to find evidence of greater trait variability in outbred mice.

Adolescence is a transitional period between childhood and adulthood and is often when alcohol use begins (Spear, 2015). During this stage, mesolimbic and frontal cortical regions undergo significant remodeling (Reynolds & Flores, 2021). The maturation state of these regions may influence the propensity for experimentation and continued drug use (Spear, 2015). Behavioral traits such as sociability, response to positive rewards, risk‐taking/novelty‐seeking behavior, anxiety, and stress‐coping abilities have been linked to drug use during adolescence (Adalbjarnardottir & Rafnsson, 2002; Comeau et al., 2001; Geier, 2013; Woicik et al., 2009). Here, adolescent mice exhibited higher novelty‐seeking behavior, displayed more activity and less anxiety; however, machine learning analyses revealed that those behaviors had a low impact on ethanol preference susceptibility. In rodents, a preference for novelty has been linked to increased sensitivity to the reinforcing effects of various drugs of abuse, including amphetamine, cocaine, nicotine, and alcohol (Nadal et al., 2002). However, the relationship between novelty seeking and alcohol intake in rodents remains unclear. Novelty seeking is often assessed in rodents through increased locomotor activity in a new environment. In outbred rats, previous studies have reported positive (Karatayev et al., 2010), negative (Gingras & Cools, 1995), or no correlation (Hayton et al., 2012) between novelty‐induced activity and alcohol consumption. Along the same lines, individual differences in anxiety do not appear to have consistent effects on ethanol reward. Data from Hayton et al. (2012) suggest that higher anxiety‐related behavior predicts alcohol intake in adult male rats (Hayton et al., 2012). Conversely, anxiety‐like behavior in adolescent male C57BL/6 mice negatively correlates with the propensity for alcohol reward in conditioned place preference (Huang et al., 2018). This variability highlights the possibility that other behavioral and neurobiological traits, which differ between models, may act as confounding factors. As a result, a more comprehensive analysis of the behavioral profile is essential for predictive studies of drug consumption. Furthermore, our data do not exclude the possibility that drastic conditions, such as the development of anxiety disorders, could contribute to altered susceptibility to the effects of ethanol and its consumption. For example, in the case of nicotine, previous work from our group demonstrated that baseline anxiety does not seem to predict the subsequent pattern of nicotine consumption in naïve mice (Abreu‐Villaça et al., 2006). However, a pathological anxiogenic response, such as that induced by drug withdrawal, may be a crucial factor (Manhães et al., Manhaes et al., 2008). In accordance, chronic psychosocial stress, which increases anxiety‐like behavior, also promotes an increase in ethanol consumption in the two‐bottle choice test (Bahi, 2013a, 2013b).

Individual differences in response to rewarding stimuli remain poorly studied, despite their relevance. Our data suggest that the response to natural reward, evaluated by sucrose preference, has a huge impact in predicting ethanol preference during adolescence. In fact, the mesolimbic dopamine pathways play a key role in processing the reinforcing effects of both natural rewards and alcohol (Volkow et al., 2017), which can be a common denominator in this predicting effect. This neurochemical pathway is still developing during adolescence, and has functional characteristics that increase susceptibility to drug abuse, including alcohol consumption (for review, see Abreu‐Villaça et al., 2017). However, it remains unclear how inherent or baseline variations in the activity of this circuit influence individual behavior and contribute to future susceptibility to alcohol. A recent study by Montgomery et al. (2023) found that hyperdopaminergic male mice exhibited a diminished neuronal response to both natural rewards and alcohol, predicting lower alcohol consumption. Taken together, these data corroborate the idea that natural reinforcers and ethanol activate similar brain circuitry and neurochemistry mechanisms. One of the neurotransmitter systems that may be involved in this process is orexin. The rewarding aspects of palatable food intake may be mediated by a neural connection between the nucleus accumbens, a crucial regulator of reward‐related behaviors, and hypothalamic orexin neurons (Zheng et al., 2007). In fact, data from Olney et al. (2017) suggest that the orexin‐1 receptor is the predominant receptor subtype within the ventral tegmental area and central nucleus of the amygdala that regulates binge‐like ethanol drinking. Accordingly, several preclinical studies demonstrated that orexin receptor antagonists reduce ethanol consumption (Lopez et al., 2016; Olney et al., 2015). In this sense, individual variations in these systems could explain the predicting value of the response to natural reward in ethanol preference. Furthermore, although not directly assessed in the present study, our results may suggest that the orexin system plays different roles in the susceptibility to ethanol preference between adolescents and adults, since the response to natural rewards was determinant only in adolescent animals. We hypothesize that orexin receptor antagonism may represent a potent mechanism for reducing ethanol use in adolescents and, therefore, may represent a potential therapeutic target for the treatment of alcohol use disorders at this age. These interpretations are speculative and should be further evaluated in future studies.

There is a strong influence of the social environment on ethanol experimentation and consumption, especially during adolescence. Studies demonstrate that one of the motivations for ethanol consumption is linked to socialization, as adolescents hold beliefs about how alcohol will positively affect or enhance social interactions (Read et al., 2003). In fact, adolescent social isolation results in changes in the dopamine system and increases ethanol consumption in rodents (Ortelli et al., 2023; Petersen et al., 2024). Additionally, adolescents appear to be more sensitive than adults to ethanol‐induced social facilitation (Varlinskaya & Spear, 2002). Our data indicated that adolescent mice with a low sociability profile show increased susceptibility to higher ethanol consumption, reinforcing the idea that pathways involved in the regulation of sociability may relate to the motivation for ethanol consumption. Given the relevance of oxytocin's effects on social behavior (Lukas et al., 2011), our findings suggest that individual variations in the function of oxytocinergic pathways might influence the reward response associated with ethanol during adolescence, contributing to the susceptibility profile observed here. In fact, oxytocin administration reduces ethanol intake in both humans and animals (for review, see Peris et al., 2020). Although studies on the role of oxytocin administration in the rewarding effects of ethanol during adolescence are limited, oxytocin administration may also be a promising therapeutic target for alcohol use disorder at this stage of development.

Interestingly, adult animals did not exhibit a behavioral profile capable of predicting subsequent ethanol preference. This finding suggests that the brain pathways primarily involved during adolescence may no longer be highly susceptible to the effects of ethanol in adulthood. This could, at least in part, explain why experimentation with ethanol in adulthood is less likely to lead to problematic consumption compared with adolescence. These differences have significant implications for therapeutic strategies aimed at preventing and treating alcohol use disorders, as they may influence the effectiveness of interventions, the selection of pharmacological treatments, and behavioral approaches.

The present study had some limitations. One important issue is that our results were obtained with a small sample size (adolescence = 46 animals, adulthood = 79 animals). It is important to emphasize that the limited number of animals could compromise the generalizability of the findings. Although we applied two different cross‐validation strategies to demonstrate the model's generalizability, the ideal scenario would involve training and testing the model with truly independent samples. Our results pave the way forward for future studies using completely independent replication samples to assess the generalizability of the proposed approach. Second is the potential confounding effect of sex and strain on behavioral data. As explained in the methods and results sections, to address this issue, strains and sex were treated as binary variables and regressed out of the pattern regression analysis using the residual forming matrix framework when decoding ethanol consumption in adolescence. The main limitation was the fact that these two potential confounders (sex and strain) were associated with the variable we wanted to predict in Model 2 (adulthood). Therefore, removing their effect from patterns of behavioral profile would also remove the variability associated with the data. In order to overcome this limitation, we employed a manual matching of animals across the different cross‐validation splits (to keep balanced proportions of sex and strain). Finally, we are aware that Pearson's correlation coefficients on their own do not provide a satisfactory way of evaluating the model's performance. For this reason, we only considered the predictive models to be significant when the mean squared error's *p*‐values (estimated using permutation tests) were also below the significance threshold of 0.05.

## CONCLUSION

Using a machine learning analysis model, we demonstrated that the behavioral profile predicts ethanol preference during adolescence. Natural reward preference and sociability strongly contribute to this prediction in adolescents and suggest the existence of neurochemical pathways associated with susceptibility to ethanol consumption at this age.

## CONFLICTS OF INTEREST

The authors declare that they have no conflicts of interest.

## Supporting information


Data S1

## Data Availability

The data that support the findings of this study are available from the corresponding author upon reasonable request.
