# Supplementary material for: Behavioral profile predicts ethanol preference in adolescent mice, but not in adults: A machine learning approach
Source: Alcohol Clin Exp Res (Hoboken). 2026 Jan 23;50(1):e70203. doi: 10.1111/acer.70203 (PMC12829523; doi:10.1111/acer.70203)
Supplement: Supplementary file 1 — Data S1 [file ACER-50-0-s001.docx]

**Supplemental Material**

**Table S1:** Measurements of agreement between the actual and estimated scores based on behavioral phenotypic profile during adolescence (Model 1) and adulthood (Model 2).

| ***Model*** | ***Algorithm*** | ***Folds*** | ***Measures of agreement*** | |
| --- | --- | --- | --- | --- |
|  |  |  | ***r (p-value)*** | ***NMSE (p-value)*** |
| Model 1  (Adolescence) | GPR | Five | 0.40 (0.01) | 0.90 (0.05) |
|  | KRR | Two | 0.48 (0.004) | 0. 74 (0.001) |
|  |  | Five | 0.40 (0.03) | 0.90 (0.05) |
| Model 2 (Adulthood) | GPR | Five | 0.23 (0.07) | 0.94 (0.05) |
|  | KRR | Two | 0.11 (0.23) | 1.08 (0.34) |
|  |  | Five | 0.22 (0.08) | 0.93(0.04) |

Significant results are displayed in red.

**Table S2:** Measurements of agreement between the actual and estimated scores based on behavioral phenotypic profile during adolescence (Model 1) for each separate fold

| ***Model 1*** | ***Folds*** | ***Measures of agreement*** | |
| --- | --- | --- | --- |
|  |  | ***r*** | ***NMSE*** |
| Adolescence  (GPR) | One | 0.51 | 0.71 |
|  | Two | 0.44 | 0.78 |
|  | All folds average | 0.47 (0.004) | 0.74 (0.001) |

**Table S3:** **Multivariate linear regression for the prediction of ethanol preference during adolescence**

| **Ethanol**  **preference** | **Beta Coefficient** | **t** | **p-value** |
| --- | --- | --- | --- |
| Sucrose Consumption | 0.46 | 3.14 | 0.004 |
| Novelty-Seeking | -0.07 | -0.54 | 0.59 |
| Anxiety | -0.005 | -0.04 | 0.97 |
| Sociability | -0.28 | -2.0 | 0.054 |
| Stress-Coping | 0.16 | 1.16 | 0.25 |

Significant results are displayed in red.

**Table S4: Multivariate linear regression for the prediction of ethanol preference during adulthood**

| **Ethanol**  **preference** | **Beta Coefficient** | **t** | **p-value** |
| --- | --- | --- | --- |
| Sucrose Consumption | -0.03 | -0.29 | 0.77 |
| Novelty-Seeking | -0.1 | -0.85 | 0.39 |
| Anxiety | -0.07 | 0.61 | 0.54 |
| Sociability | 0.14 | 1.16 | 0.25 |
| Stress-Coping | 0.25 | 2.8 | 0.10 |

**Comparison between models (adolescence × adulthood).**

We performed additional analyses to statistically compare the two models (adolescence and adulthood), we employed a Wilcoxon rank-sum (Mann-Whitney) to compare the distribution of r and NMSE obtained in each fold for both algorithms. Interestingly, both metrics are statistically different (r (z-score=2.34, p-value=0.01), NMSE (z-score=-2.32, p-value=0.02)) in the two fold-cross validation indicating that they are themselves different. One limitation of these results is that we have small numbers of r and NMSE observations. Further investigation with a larger sample comparing the two models (adolescence and adulthood) directly is necessary.
